# Association of Maximum Weight with Hyperuricemia Risk: A Retrospective Study of 21,414 Chinese People

**DOI:** 10.1371/journal.pone.0051186

**Published:** 2012-11-30

**Authors:** Bin Gao, Jie Zhou, Jiapu Ge, Yaping Zhang, Fei Chen, Wayne B. Lau, Yi Wan, Nanyan Zhang, Ying Xing, Li Wang, Jianfang Fu, Xiaomiao Li, Hongxia Jia, Xin Zhao, Qiuhe Ji

**Affiliations:** 1 Department of Endocrinology, The First Affiliated Hospital of the Fourth Military Medical University, Xi'an, Shaanxi, China; 2 Department of Endocrinology, Xinjiang Uger Autonamy People's Hospital, Urumuqi, Xinjiang, China; 3 Department of Endocrinology, The First Affiliated Hospital of the Second Military Medical University, Shanghai, China; 4 Department of Emergency Medicine, Thomas Jefferson University, Philadelphia, Pennsylvania, United States of America; 5 Department of Health Statistics, The Fourth Military Medical University, Xi'an, Shaanxi, China; Gentofte University Hospital, Denmark

## Abstract

**Background:**

Obesity has been demonstrated to be associated with increased serum uric acid (SUA); however, little is known regarding the relationship between maximum weight, or maximum weight fluctuation, and uric acid concentration. Through retrospective means, we determined the association of maximum weight with SUA risk.

**Methods:**

Data of 21,414 participants (8,630 males and 12,784 females) from the 2007-8 China National Diabetes and Metabolic Disorders Study were analyzed for parameters including lifestyle habits, biochemical blood analysis and self-reported maximum weight.

**Results:**

Elevated SUA subjects shared a cluster of demographic features. After adjustment for age, gender, education, smoking, drinking, physical activity, WHR, height, eGFR(evaluate glomerular filtration rate), and diuretic usage, multivariate logistic regression models demonstrated maximum weight was associated with increased risk of elevated SUA level (P<0.001). Duration of maximum weight was related with decreased risk of elevated SUA level (P<0.001). There was a significant correlation between time of weight loss and risk of increased SUA level reduction (P<0.001). Furthermore, our data indicated that the degree of weight loss from maximum weight was another important factor for the risk of increased SUA level reduction (P<0.001). Finally, ROC curve analysis revealed area under the curve was 0.661 (95% CI, 0.647-0.674), statistically significant for maximum weight association with hyperuricemia (P<0.001).

**Conclusions:**

Maximum weight is a strong risk factor for increased uric acid level in the Chinese population, which might serve as a novel clinical indicator suggesting hyperuricemia. Controlling maximum weight, keeping weight to the appropriate range, and maintaining the stable weight may be conducive for decreasing risk of hyperuricemia.

## Introduction

Worldwide prevalence of hyperuricemia is increasing rapidly. Data from a large managed care database in the USA indicates that the annual prevalence of gout and/or clinically significant hyperuricemia increased from 2.9 per thousand in 1990 to 5.2 per thousand in 1999[1]. In the coastal city Tianjin of China, hyperuricemic prevalence was 12.16%, with male significantly higher than female in 2011[2]. Increasing evidence supports a relationship between hyperuricemia and metabolic syndrome risk factors including hypertension, hyperlipidemia, diabetes, obesity, and insulin resistance [3], [4], [5], [6]. Also, many studies have focused on the association between serum uric acid (SUA) and weight. After age adjustment, gout patients have significantly greater body mass index (BMI) in the Framingham Study [7]. After 2-year follow up of 3,153 individuals, Ishizaka reported BMI change was a predictor for SUA change [8]. As the association between obesity and SUA is well established, weight is an important modifiable risk factor for hyperuricemia [8], [9].

Weight loss is not a simple, nor uniform, matter in different persons. Despite efforts, many obese individuals have difficulty altering their overweight status, and many enter a cyclical pattern of weight loss with re-gain [10]. Continued aerobic exercise post weight re-gain may counter the detrimental effects of partial weight re-gain, as evidenced by several metabolic markers [11]. Weight fluctuations in the obese condition are therefore closely associated with metabolic disorders. Several large population-based epidemiologic studies of diabetes mellitus have investigated maximum weight, reflective of the maximum obesity state [12], [13]. However, data remains limited on the association between maximum weight and SUA, or regarding weight change from the maximum obesity condition. The 2007–8 China National Diabetes and Metabolic Disorders Study is the most current national cross-sectional survey in China [14]. Drawing from the survey database, we analyzed the association of the risk of high SUA level with maximum weight, weight stability, time of weight loss and the degree of weight loss from the maximum weight. This study maybe have potential clinical application of assessing hyperuricemia risk in the obese population.

## Methods

### Study population

All data analyzed in the present study originated from the 2007–8 China National Diabetes and Metabolic Disorders Study, a cross-sectional study that obtained data from June 2007 to May 2008 via a multi-stage, stratified sampling design. Details regarding its sampling methods were based upon our group's previous study [14]. SUA was not a requisite test item in each region in our previous study, so 35 cities and 19 countries evaluated SUA. A total of 22,020 people (9,120 males and 12,900 females) were included into our database analysis. Additionally, 376 subjects were excluded from the study due to incomplete information regarding self-reported maximum weight, and 230 subjects were excluded due to missing SUA data. Ultimately, 21,414 subjects were analyzed in the present study. In addition, 50 subjects lacked the information of smoking and drinking. So these people had not included in the analysis of Table 1. All subjects had been in our previous study signed an informed consent.

**Table 1:** Study Cohort Characteristics per SUA Quartile.

Characteristics	Male quartile of Uric Acid Concentration^a^(N = 8630)			
	1(<265µmol/L)	2(265µmol/L-317µmol/L)	3(317µmol/L-374µmol/L)	4(>374µmol/L)
No. of subjects	2160	2146	2190	2134
Mean uric acid (95% CI), µmol/L	221.47(219.91–223.03)	292.19(291.55–292.82)	344.68(343.99–345.37)	436.52(434.03–439.02)
Mean creatinine (95% CI), µmol/L	81.80(80.64–82.96)	83.24(81.05–85.42)	83.02(81.77–84.28)	86.48(85.24–87.71)
Mean age (95% CI)^b^, y	45.78(45.18–46.39)	44.92(44.32–45.53)	44.92(44.33–45.51)	44.98(44.38–45.58)
Smoking (95% CI)^c^, %	49.16(48.02–52.24)	52.33(50.22–54.44)	53.34(50.41–54.65)	54.53(52.25–56.43)
Drinking (95% CI)^d^,%	37.87(35.81–39.90)	46.32(44.21–48.43)	49.45(47.36–51.55)	55.25(53.14–57.36)
Mean current weight(95% CI), Kg	64.46(64.02–64.88)	67.27(66.80–67.73)	69.43(68.96–69.89)	73.68(73.16–74.20)
Mean BMI (95% CI), kg/m^2^	23.02(22.88–23.15)	23.86(23.70–24.01)	24.46(24.31–24.61)	25.78(25.62–25.94)
Mean WC (95% CI), cm	82.17((81.76–82.58)	83.91(83.48–84.34)	85.65(85.22–86.07)	89.32(88.88–89.76)
Mean WHR (95% CI)	0.87(0.86–0.87)	0.88(0.87–0.88)	0.89(0.88–0.89)	0.91(0.90–0.91)
Mean maximum weight (95% CI), Kg	69.13(68.66–69.61)	71.75(71.22–72.27)	73.26(72.75–73.77)	77.26(76.73–77.79)
Mean age at maximum weight (95% CI), y	35.32(34.73–35.91)	36.84(36.25–37.42)	37.20(36.61–37.79)	38.54(37.95–39.12)
Mean maximum BMI (95% CI), kg/m^2^	24.70(24.55–24.85)	25.46(25.28–25.63)	25.81(25.65–25.98)	27.04(26.87–27.19)
Mean Δweight (95% CI), Kg	4.68(4.41–4.94)	4.48(4.18–4.78)	3.83(3.57–4.09)	3.58(3.38–3.78)

	Female quartile of Uric Acid Concentration^a^(N = 12784)			
	1(<199µmol/L)	2(199µmol/L-238µmol/L)	3(238µmol/L-282µmol/L)	4(>282µmol/L)
No. of subjects	3232	3239	3111	3202
Mean uric acid (95% CI), µmol/L	166.68(165.70–167.65)	219.35(218.95–219.74)	259.17(258.73–259.61)	333.51(331.62–335.40)
Mean creatinine (95% CI), µmol/L	65.60(64.96–66.23)	65.73(65.11–66.35)	68.53(64.10–72.95)	70.58(69.07–72.10)
Mean age (95% CI), y	42.33(41.91–42.75)	43.34(42.89–43.79)	45.32(44.86–45.79)	48.64(48.15–49.13)
Smoking (95% CI)^bc^, %	2.51(1.97–3.05)	2.41(1.88–2.94)	2.96(2.36–3.55)	3.34(2.72–3.96)
Drinking (95% CI)^bd^, %	3.84(3.17–4.50)	4.38(3.68–5.09)	4.50(3.77–5.23)	5.18(4.42–5.95)
Mean current weight (95% CI), Kg	56.20(55.91–56.49)	57.45(57.15–57.76)	59.34(59.00–59.68)	61.60(61.23–61.96)
Mean BMI (95% CI), kg/m^2^	22.76(22.65–22.87)	23.25(23.13–23.36)	24.02(23.89–24.15)	24.95(24.81–25.09)
Mean WC (95% CI), cm	76.66(76.33–76.97)	77.68(77.34–78.01)	79.64(79.28–80.00)	82.37(81.99–82.74)
Mean WHR (95% CI)	0.82(0.81–0.83)	0.83(0.82–0.83)	0.84(0.83–0.84)	0.86(0.85–0.86)
Mean maximum weight (95% CI), Kg	60.44(60.11–60.76)	61.63(61.29–61.97)	63.33(62.96–63.69)	65.30(64.93–65.68)
Mean age at maximum weight (95% CI), y	33.55(33.11–33.99)	34.93(34.46–35.39)	37.27(36.77–37.76)	39.83(39.3–40.36)
Mean maximum BMI (95% CI), kg/m^2^	24.48(24.35–24.61)	24.94(24.81–25.07)	25.63(25.49–25.77)	26.45(26.20–26.59)
Mean Δweight (95% CI), Kg	4.24(4.04–4.43)	4.17(3.96–4.38)	3.98(3.77–4.20)	3.69(3.51–3.88)

Continuous data are presented as mean, Categorical data are presented as percentage. To convert uric acid level from μmol/L per liter to mg/dL, values were divided by 59.48. BMI, body mass index; WC, waist circumference; WHR, waist to hip ratio; Δweight: difference between current and maximum weight. Maximum weight could be the current weight.

a : All *P* values <0.001.

b : *P* values >0.05.

c : Smoking was defined as having smoked at least 100 cigarettes in one's lifetime.

d : Drinking was defined as consumption of at least 30 g of alcohol per week for 1 year or more.

### Data collection

In the 2007–8 China National Diabetes and Metabolic Disorders Study, a physical examination was performed on all subjects by a qualified doctor per established standard methods [15]. This physical examination documented measurements of height, weight, waist circumference (defined as smallest circumference between the rib margin and iliac crest), hip circumference (defined as maximum circumference between the waist and hips). The accuracy of all above parameters is defined as 0.5 cm. Body mass index (BMI) was calculated by dividing body weight (kg) by the square of height (m2). Maximum BMI was calculated by dividing maximum body weight (kg) by the square of height (m2). Waist-to-hip ratio (WHR) was calculated as waist circumference divided by hip circumference. Data collection was performed at local health stations or community clinics. A standardized questionnaire was completed by trained physicians and nurses. Data collected from each subject included lifestyle, self-reported maximum weight, medication usage and educational level. Smoking was defined as smoking one or more cigarettes daily for at least one year. Alcohol use was defined as consumption of at least 30 grams of alcohol per week for 1 year or more. Regular leisure-time physical activity was defined as participation in moderate or vigorous activity for 30 minutes or more daily at least 3 days per week. Maximum weight was defined as, heretofore presently, the maximum weight the subject has attained, as self-reported. Duration of maximum weight was defined as, to the survey date, the subject self-reported time that maximum weight kept stable. Time interval between maximum and current weight was defined as time from the end of self-reported maximum weight (essentially time of weight loss took place) to the survey date. Absolute weight loss was defined as the difference between maximum weight and current weight. If the maximum weight was the current weight, the difference would be zero. Hyperuricemia was defined as SUA concentration>7.0 mg/dl (420 µmol/l, males) or 6.0 mg/dl (357 µmol/l, female) as previously defined [16], [17].

After physical examination, participants were subject to collected fasting venous blood specimen. Fasting serum was utilized to determine uric acid and other serum biochemical parameters. All biochemical testing had successfully completed at a standardization/certification program.

### Statistical Analysis

EpiData (EpiData Association, Odense, Denmark) established the database, and SPSS16.0 for Windows (SPSS Inc, Chicago, IL, USA) was utilized for statistical analyses. After statistical analysis, all variables were normal distribution and presented either as mean for continuous variables or proportions for categorical variables. Comparisons among groups were tested by one-way ANOVA. The chi-squared test was employed to estimate the difference for categorical variables. Partial correlation analysis determined the relationship between SUA and related variables. Multivariate logistic regression models examined the association between maximum weight and concentrations of SUA, after adjusting for age, sex, height, smoking, drinking, education, physical activity, WHR, eGFR (eGFR = 186× Scr (mg/dl)^−1.154^× age (years)^−0.203^(×0.742, if female)) [18], and diuretic use. All statistical tests were two-tailed with type I error set at 0.05, and *P* values <0.05 considered statistically significant.

## Results

21,414 participants (8,630 males and 12,784 females) were included in our final analysis (Table 1). Subjects of the highest SUA quartile shared a cluster of demographic features. Based on our data, male showed higher SUA concentration and their likelihood of smoking and consuming alcohol increased dramatically. Table 1 listed the weight related variable characteristics of the different SUA quartiles. Current weight, BMI, waist circumference and WHR were associated with increased SUA (P<0.001). Both self-reported maximum weight and BMI increased with elevated SUA. Age at maximum weight was increased with elevated SUA (P<0.001). Discrepancy between current and maximum weight was diminished with elevated SUA level. Age- and gender-adjusted partial correlation analysis demonstrated a statistically positive correlation between uric acid level and maximum weight, maximum BMI, and age at maximum weight, correlation coefficient were 0.194, 0.177, and 0.104, respectively.

After adjustment for the confounding variable of age, gender, education, smoking, drinking, physical activity, WHR, height, eGFR and diuretic usage, maximum weight was associated with increased risk of elevated SUA level (Table 2). When maximum weight exceeded 70 kilograms, multiple logistic regression models revealed the odds ratio (OR) of concomitant elevated SUA level was 1.275, 2.233, and 3.513 in the second, third, and fourth SUA quartile respectively compared to the lowest SUA quartile. After adjustment for the same confounding variables, the duration of maximum weight was associated with reduced risk of elevated SUA levels (Table 3). For duration of maximum weight extending beyond 3 years, compared to the lowest SUA quartile, the OR in the second, third, fourth quartile was 0.619, 0.498, and 0.410, respectively. Analysis of the interval time between maximum and current weight (essentially time of weight loss took place) demonstrated the longer time interval with the lower risk of uric acid elevation (Table 4), particularly when the time interval exceeded 10 years (OR for 0–5 year duration, 5–10 year duration and >10 year duration in the highest SUA quartile was 0.615, 0.505, and 0.341, respectively). Finally, multivariate logistic regression models demonstrated significant risk reduction for elevated SUA as maximum weight loss increased (Table 5). When absolute maximum weight loss exceeded 5 kilograms, the OR in the second, third, and fourth quartile was 0.682, 0.522, and 0.407, in comparison to the lowest SUA quartile.

**Table 2:** Multiple Logistic Regression Analyses of SUA quartiles Association with Maximum Weight.

Uric Acid Quartile	Maximum Weight (OR 95%CI)		
	<60Kg	60–70Kg	>70Kg
Quartile 1	1.000	1.000	1.000
Quartile 2	1.000	1.109(0.976–1.261)	1.275(1.076–1.509)
Quartile 3	1.000	1.447(1.263–1.657)	2.233(1.882–2.648)
Quartile 4	1.000	1.664(1.423–1.944)	3.513(2.916–4.232)

This model was adjusted for age, sex, height, smoking, drinking, education, physical activity, waist-hip ratio, eGFR, and diuretic use. CI, Confidence interval; OR, odds ratio; eGFR, evaluate glomerular filtration rate.

**Table 3:** Multiple Logistic Regression Analyses of SUA Quartiles Association with Duration of Maximum Weight.

Uric Acid Quartile	Duration of Maximum Weight (OR 95%CI)				
	0year	0–1 year	1–2 year	2–3 year	>3 year
Quartile 1	1.000	1.000	1.000	1.000	1.000
Quartile 2	1.000	0.783 (0.650–0.942)	0.754 (0.638–0.891)	0.634 (0.510–0.787)	0.619 (0.518–0.739)
Quartile 3	1.000	0.673 (0.568–0.799)	0.579 (0.477–0.702)	0.537 (0.448–0.643)	0.498 (0.398–0.623)
Quartile 4	1.000	0.577 (0.481–0.693)	0.509 (0.414–0.626)	0.423 (0.349–0.513)	0.410 (0.323–0.521)

This model was adjusted for age, sex, height, smoking, drinking, education, physical activity, waist-hip ratio, eGFR, and diuretic use. CI, Confidence interval; OR, odds ratio. eGFR, evaluate glomerular filtration rate.

**Table 4:** Multiple Logistic Regression Analyses of SUA Quartiles Association with Time Interval between the end of Maximum and Current Weight.

Uric Acid Quartile	Time interval between the end of maximum and current weight (OR 95%CI)			
	0 year	0–5 year	5–10 year	>10 year
Quartile 1	1.000	1.000	1.000	1.000
Quartile 2	1.000	0.855 (0.730–1.000)	0.668 (0.543–0.821)	0.596 (0.493–0.721)
Quartile 3	1.000	0.686 (0.583–0.807)	0.562 (0.453–0.696)	0.384 (0.313–0.470)
Quartile 4	1.000	0.615 (0.516–0.733)	0.505 (0.398–0.639)	0.341 (0.274–0.426)

This model was adjusted for age, sex, height, smoking, drinking, education, physical activity, waist-hip ratio, eGFR, and diuretic use. CI, Confidence interval; OR, odds ratio. eGFR, evaluate glomerular filtration rate.

**Table 5:** Multiple Logistic Regression Analyses of SUA Quartiles Association with Absolute Weight Loss.

Uric Acid Quartile	Degree of Weight Loss (OR 95%CI)			
	0Kg	0–2Kg	2–5Kg	>5Kg
Quartile 1	1.000	1.000	1.000	1.000
Quartile 2	1.000	0.837 (0.715–0.979)	0.692 (0.597–0.802)	0.682 (0.587–0.791)
Quartile 3	1.000	0.702 (0.597–0.824)	0.592 (0.509–0.688)	0.522 (0.448–0.609)
Quartile 4	1.000	0.738 (0.621–0.876)	0.554 (0.471–0.652)	0.407 (0.344–0.481)

This model was adjusted for age, sex, height, smoking, drinking, education, physical activity, waist-hip ratio, eGFR, and diuretic use. CI, Confidence interval; OR, odds ratio. eGFR, evaluate glomerular filtration rate.

**Figure 1:**
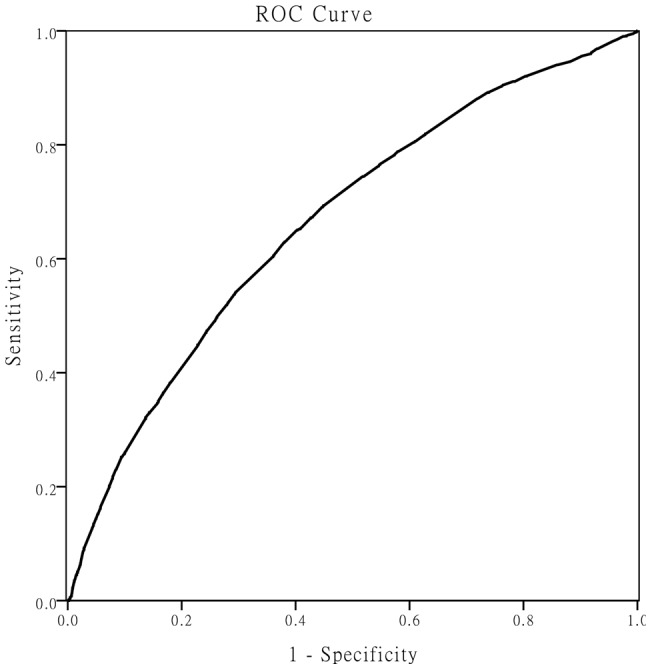
ROC curve demonstrating diagnostic value of maximum weight forhyperuricemia. Receiver operating characteristic (ROC) curve analysis demonstrated statistically significant value of maximum weight for hyperuricemia diagnosis (area under the curve was 0.661 with 95% CI of 0.647–0.674, P<0.001).

Receiver operating characteristic (ROC) curve analysis demonstrated statistically significant value of maximum weight for hyperuricemia diagnosis (area under the curve was 0.661 with 95% CI of 0.647–0.674, P<0.001). The sensitivity and specificity for a maximum weight value 70 kilograms in diagnosing hyperuricemia was 53.5% and 70.4%, respectively (Figure 1).

## Discussion

In this retrospective study, we have demonstrated the relationship between the risk of high SUA level and maximum weight in the Chinese population. Especially in the group of subjects with high SUA level, their maximum weight and maximum BMI were usually high. After adjustment for related confounding factors, multivariate logistic regression models revealed an association between maximum weight and increased risk of SUA elevation. Weight stability, time of weight loss, the degree of weight loss, and weight loss itself were inversely related with risk of SUA elevation. Finally, ROC curve analysis demonstrated a statistically significant diagnostic value of maximum weight for hyperuricemia diagnosis.

Although several studies have previously demonstrated association between SUA and body weight [19], [20], the relationship between maximum individual body weight and the risk of hyperuricemia was unknown. To our knowledge, the present study is the first reporting the association of maximum weight with increased risk of elevated SUA level. Maximum weight is thought to be a more valuable prediction marker for the risk of elevated SUA level. For the clinical doctors, maximum weight is more attractive for it is easy to get it. So doctors are easier to predict the individuals' risk of high SUA. Especially for those individuals whose maximum weight are over 70 kg, doctors need remind them to be followed up (Table 2, Figure 1).

Previous studies often chose maximum BMI as an indicator for high SUA level. Here we used maximum weight not BMI. We think maximum weight can minimize the potential deviation from the height to some extent. Strictly speaking, for maximum BMI, height can be used only at the time when subjects' weights reach the maximum value. In our survey, the percentage of the seniors is 16.18% of the over all participants. For these individuals, it is too difficult to recall the exact height when the weight was at the highest values in lifetime. And in fact the BMI values change as a result of shrink in height with the aging process [21], [22]. In addition, we also found that for correlation coefficient, maximum BMI or maximum weight were 0.177 and 0.194 respectively. The area under the ROC curve of maximum BMI and maximum weight was 0.637 (data not shown) and 0.661 sequentially. These data indicated that maximum weight is slightly superior to maximum BMI in our study.

Next, we further analyzed sub-parameters including time of weight loss and the degree of weight loss from the maximum weight. In this study, we found that weight loss from maximum weight is more significant to decrease the risk for SUA elevation. Previous published paper indicated that weight loss was thought to be an effective non medical strategy for SUA level reduction in the Japanese population [8]. Men losing 10 pounds or more had a 39% lower risk of developing gout [23]. But it is still necessary to observe the effects of weight loss, especially from maximum weight on SUA levels with large-scale study in Chinese population. Our data suggested that the risk of high SUA level became lower with weight loss increasing. Moderate weight loss (<5 Kg)lower the risk of SUA elevation up to 45%. Over 5 Kg weight loss will further reduce the risk by 60% at least (Table 5). So we think that more pounds you lose, more benefits to reduce risk of hyperuricemia you get, especially for individuals who lose weight over 5 kilograms from maximum weight.

Furthermore, the time of weight loss starting was thought to be another important factor. Ezequiel DG et al. reported weight reduction group showed a trend toward a reduction of SUA after a 12-week calorie-restricted diet [24]. Another study that observation of obese who treatment with sibutramine for 1 year showed serum uric acid was lowered significantly with weight loss [25]. But it is still not clear how the time of weight loss starting, or time interval from the end of self-reported maximum weight to the survey date influents SUA elevation. We have strong evidence that the longer interval, the lower risk of SUA level elevation (Table 4). That means individuals need lose weight as early as possible from the maximum weight. That definitely will bring more benefits to lower risk of hyperuricemia and its consequences.

During the process of analyzing data, we found in all of loosing weight subjects, the percentage was 43% whose weight went down to their standard weight from maximum weight. The rest subjects show weight loss with different degrees. For some reasons such as psychological factors and living habits, it is too difficult for them to lose weight continuously. So at this time, keeping weight stability or rapid weight fluctuation, which one is better for decreasing their risk of high SUA level? As we all known, weight fluctuation (loss-gain or gain-loss) can alter various metabolic factors such as blood pressure, blood sugar, SUA, high-density-lipoprotein-cholesterol and obesity [26]. When subjects experience weight fluctuations, SUA level will be changed as a result, although rapid weight loss would elevate uric acid level temporarily [27]. The ERFORT Male Cohort Study also indicated that weight fluctuations are a major risk factor for all-cause mortality. Stable weight does not increase further mortality even if they are obese [28]. On this point, we got the similar results. Our study demonstrated maintaining weight stability will be beneficial for risk of high SUA reduction (Table 3). Of course, loosing weight firstly appears to have more positive effects upon reduce risk of uric acid elevation. So based on above mentioned, we propose that individuals should attempt to maintain a stable weight especially for them whose weights are too difficult to keep loosing continuously, no matter their weights go down to standard range.

The Framingham Heart Study established the dangerous relationship between SUA level elevation and coronary heart disease occurrence, cardiovascular death, and all-cause mortality in females [7]. An eight years follow-up study upon 128,569 adults conducted by Pan in Taiwan, China, concluded the hyperuricemia was independently associated with the development of ischemic heart disease not only in the general population but also in those without any metabolic risk factor [29]. The extension of our study's results demonstrating weight stability with decreased risk of SUA level elevation to actual decreased risk of cardio/cerebrovascular events is under current investigation.

Our study has several limitations. Firstly, we have no information regarding the extent to which lifestyle and dietary habit modifications affect our study population. Secondly, information regarding body maximum weight was self-reported, whereas current weight and height were directly measured by trained staff. Previous studies, however, have validated self-reported past body weight, demonstrating great accuracy in comparison to measured body weight [30], [31]. Thirdly, our study's data set was a part of the 2007–8 China National Diabetes and Metabolic Disorders Study, which did not assess the prevalence of hyperuricemia in China. Finally, as our study was observational, we cannot rule out the possibility unmeasured factors may contribute to observed associations.

### Conclusion

Our large retrospective study indicates that maximum weight is a strong risk factor for hyperuricemia in the Chinese population. Weight stability, time of weight loss, the degree of weight loss, and weight loss itself was inversely proportional to the risk of elevated uric acid level. Controlling maximum weight, keeping weight to the appropriate range, and maintaining the stable weight may be conducive for decreasing risk of hyperuricemia. Maximum weight is an easy and accurate indicator to value the increased SUA level, which may potentially serve as a novel clinical indicator for identifying patients at high risk of hyperuricemia.
